# Adenomatoid Hyperplasia Requiring Differential Diagnosis From Malignant Neoplasms: A Report of Two Cases

**DOI:** 10.7759/cureus.91117

**Published:** 2025-08-27

**Authors:** Taku Kimura, Takumi Shimura, Yasushi Kato, Aya Yanagawa-Matsuda, Kazuhiro Kawamura, Zen-ichi Tanei, Shinya Tanaka, Ken-ichiro Sakata

**Affiliations:** 1 Department of Oral Diagnosis and Medicine, Hokkaido University, Sapporo, JPN; 2 Department of Vascular Biology and Molecular Pathology, Faculty and Graduate School of Dental Medicine, Hokkaido University, Sapporo, JPN; 3 Department of Diagnostic Pathology, Oita University, Oita, JPN; 4 Department of Cancer Pathology, Hokkaido University, Sapporo, JPN

**Keywords:** differential diagnosis, excisional biopsy, multimodal imaging approach, oral cavity malignancies, salivary gland diseases

## Abstract

Adenomatoid hyperplasia (AH) of the minor salivary glands is a rare, benign hyperplastic lesion that typically arises in the oral mucosa, most commonly in the hard or soft palate. AH generally presents as a painless mass covered with normal mucosa. AH can mimic the clinical course of malignant salivary gland tumors, making diagnosis challenging. This series presents two cases of AH arising in unusual sites, the buccal mucosa and sublingual caruncle, which were initially suspected of being malignant tumors and were evaluated using multimodal imaging. Patient 1, a 76-year-old female, presented with a buccal mass of 6 × 8 mm in size that increased in size over time and was eventually diagnosed with AH with sialadenitis. Patient 2, an 85-year-old male, presented with an indurative mass of 4 × 4 mm in size near the sublingual caruncle, later diagnosed with AH along with sialadenitis. Both patients underwent excisional biopsy with a safety margin comparable to that for malignant tumors. Each patient is now being monitored regularly without recurrence. Our patients underwent multiple imaging modalities prior to the surgery; however, they underscored the limited utility, especially in evaluating small lesions. Therefore, histopathological examination remains crucial for its definitive diagnosis. AH can present in unusual locations and mimic malignancy during its clinical course, especially when accompanied by inflammation, emphasizing the importance of its differential diagnoses. Through our case series, we highlighted that excisional biopsy is an effective strategy, and histopathological examination is essential for accurate diagnosis.

## Introduction

Adenomatoid hyperplasia (AH) of the minor salivary gland is a rare hyperplastic lesion arising from the oral mucosa, commonly observed in the hard or soft palate [1]. AH generally presents as a mass covered with normal oral mucosa, exhibiting an indolent growth pattern, and typically remains asymptomatic. Its risk factors are still unknown; however, chronic local trauma due to denture wearing is reported to contribute to the development of the lesion [1,2]. Histopathologically, AH exhibits hyperplasia of the salivary gland, characterized by mucus acini aggregates and numerous ducts, surrounded by a stroma composed of fibrous connective tissue and inflammatory cells [2]. AH requires differential diagnosis from malignancies, as it can be difficult to distinguish clinically from malignant salivary gland tumors. Accordingly, AH has been described as a "sheep in wolf’s clothing" [3,4]. Thus far, the prevalence of benign and malignant salivary gland tumors is reported to be 65% and 35%, respectively. Both types of tumors tend to arise in the major salivary glands, including the parotid, submandibular, and sublingual glands [5]. Notably, more than 50% of minor salivary gland tumors are malignant [6]. Therefore, since AH generally develops in the minor salivary gland, it is essential to distinguish AH from malignant tumors. Excisional biopsy is the recommended treatment in AH patients, with its recurrence being rare or not expected [7,8]. In this case series, we present patients with AH developing at unusual sites, including the buccal mucosa and sublingual caruncle, who also exhibited features that mimicked malignancies, necessitating a differential diagnosis from malignant tumors.

## Case presentation

Patient 1

**Figure 1:**
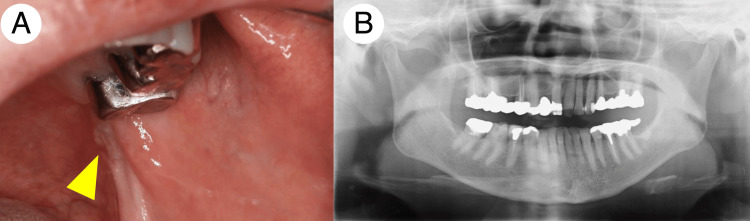
Initial clinical and panoramic X-ray findings of Patient 1 A mass lesion measuring 6 × 8 mm in size covered with a white patch was found in the left pterygomandibular fold (A). The arrowhead indicates the location of the mass lesion. A panoramic X-ray showed no apparent osteolytic lesions at the site of the mass lesion (B).

A 76-year-old female patient was referred to the Department of Oral Diagnosis and Medicine in Hokkaido University Hospital to examine a mass in her left buccal mucosa. She had a medical history of hypertension, gastroesophageal reflux disease, vasospastic angina, and diabetes mellitus. The mass lesion was pointed out by her family dentist and had remained asymptomatic until her first visit to our department. She had received a treatment with topical corticosteroid ointment for several weeks, which did not affect the lesion size. The extraoral examination revealed a mobile and non-tender lymph node on each side of her submandibular region. Her maximum mouth opening was measured at 48 mm with interincisal distance. The intraoral examination found a mass lesion measuring 6 × 8 mm near her left pterygomandibular fold (Figure 1A).

**Figure 2:**
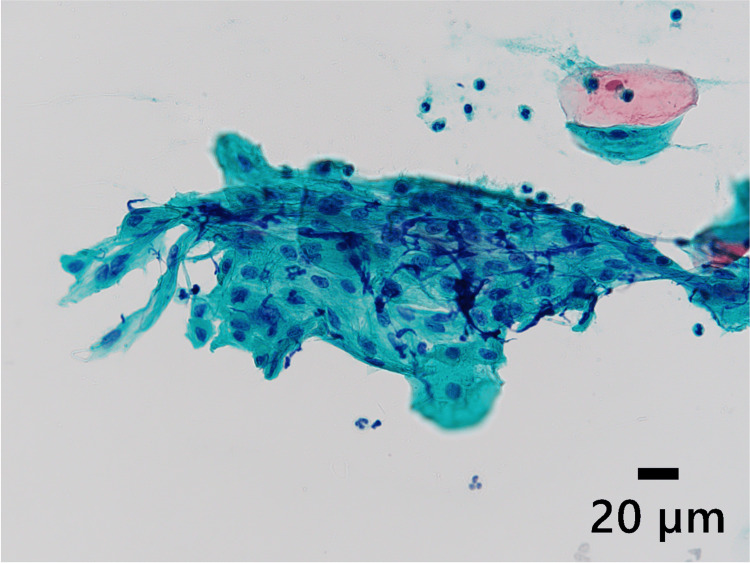
Scrape cytology result of Patient 1 Scrape cytology revealed an atypical cell cluster with mild nuclear enlargement, nuclear atypia, and small nucleoli (Papanicolaou staining, original magnification × 40).

The lesion exhibited a rough surface with soft and elastic consistency and mild tenderness on palpation. The lesion did not show a tendency to bleed. Panoramic X-ray did not reveal apparent bone loss around the lesion (Figure 1B). Scrape cytology revealed a small number of atypical cell clusters with mild nuclear enlargement, nuclear atypia, and small nucleoli, consistent with the presence of atypical cells (Figure 2).

**Figure 3:**
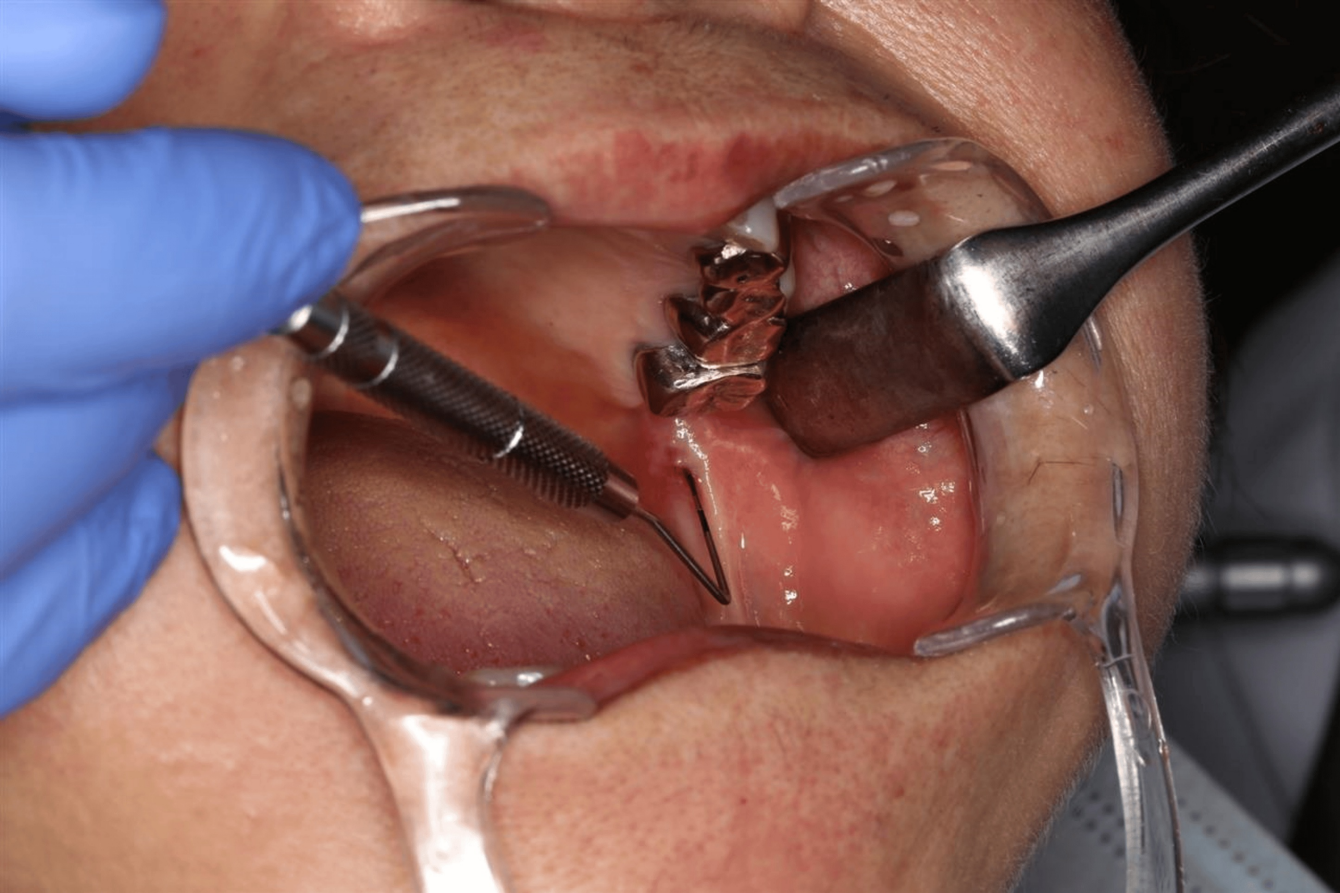
Patient 1 developed intraoral fistula in the mass lesion during the regular monitoring Five months after the first visit, the mass lesion was larger in size, at 10 × 8 mm, and accompanied by fistula formation.

Based on these examinations, the initial clinical diagnosis was a salivary gland tumor in the left pterygomandibular fold. As she was asymptomatic, the decision was made to manage the lesion with careful observation. Five months after her initial visit, she returned to our department due to discomfort while gargling. The lesion was slightly larger than it was five months ago, measuring 10 × 8 mm. The intraoral examination also identified a fistula with a depth of 5 mm (Figure 3).

**Figure 4:**
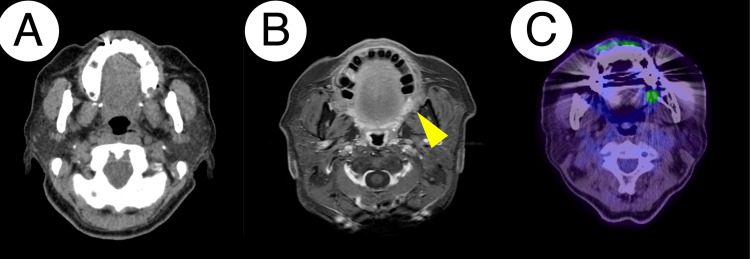
CT, MRI, and PET images of Patient 1 Contrast-enhanced CT showed no apparent enhancing mass lesion around the left pterygomandibular fold nor apparent lymph node enlargement (A). MRI revealed no abnormal findings around the lesion on the T1-weighted image, whereas the lesion displayed intermediate signal intensity (arrowhead) on the T2-weighted image (B). PET depicted a hypermetabolic lesion with its standard uptake value max of 5.128 (C). CT: computed tomography, MRI: magnetic resonance imaging, PET: positron emission tomography

There was no pus discharge from the fistula. At this time, contrast-enhanced CT and MRI revealed no significant lymph node enlargement, and the T2-weighted image displayed an intermediate signal intensity around the lesion (Figure 4A-4B).

**Figure 5:**
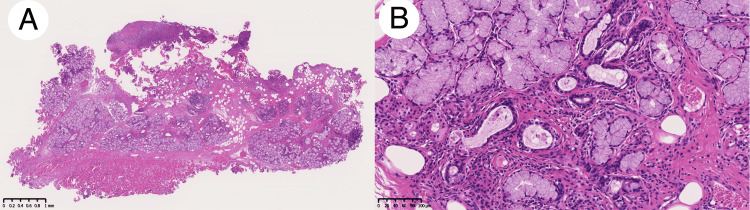
Pathological findings of Patient 1 Pathological examination revealed an increased mucous acinus and ductal proliferation accompanied by ductal dilatation and inflammatory cell infiltration (hematoxylin-eosin stain). Loupe image of the lesion (A). High magnification of the lesion (original magnification × 200).

PET/CT displayed increased fluorodeoxyglucose (FDG) uptake around the lesion with a maximum standard uptake value (SUVmax) of 5.128 (Figure 4C). Consequently, the decision was made to perform surgery with an adequate safety margin under general anesthesia, based on the suspicion of malignancies. Six months after her first visit, she underwent surgical resection with a 5 mm safety margin from the elastic area. The extent of resection reached the level of the surface of the medial pterygoid muscle. The buccinator muscle was partially included in the resected area. No ductal structure was identified during the surgery. The wound was covered with a tie-over dressing. Pathological examination revealed an increased number of mucous acini with ductal proliferation without apparent cellular atypia, accompanied by fibrosis around the mucus acini and ducts, leading to a diagnosis of AH with sialadenitis (Figure 5).

Postoperatively, she experienced trismus with a maximum mouth opening of 18 mm. Her rehabilitation relieved her symptoms, finally reaching the maximum mouth opening of 46 mm two months after the surgery. She is now under follow-up every six months without experiencing recurrence.

Patient 2

**Figure 6:**
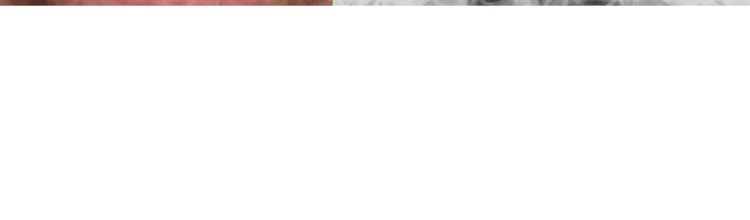
Initial clinical and panoramic X-ray findings of Patient 2 An indurative mass lesion measuring 4 × 4 mm in size with rough-surfaced mucosa developed near the sublingual caruncle (A). A panoramic X-ray showed no apparent osteolytic lesions at the site of the mass lesion (B).

An 85-year-old male was referred to the Department of Oral Diagnosis and Medicine in Hokkaido University Hospital to evaluate a lesion in the floor of his mouth. He had a medical history of hypertension and lumbar spinal stenosis. He was pointed out the lesion by his family dentist two months prior to his first visit to our department. The extraoral examination revealed a single, mobile, and non-tender lymph node on each side of his submandibular region. The intraoral examination identified a mass lesion with a 4 × 4 mm near the sublingual caruncle (Figure 6A).

**Figure 7:**
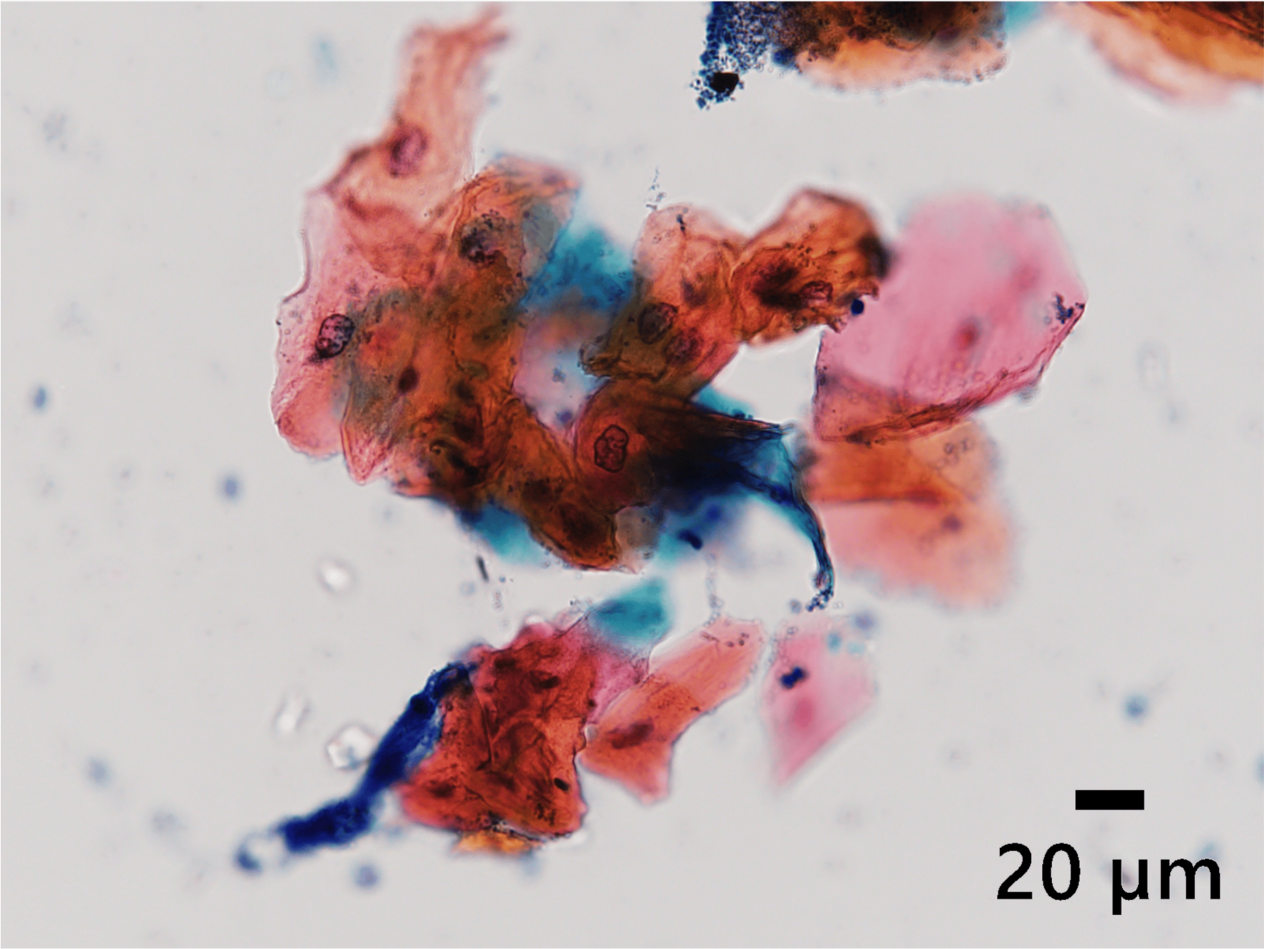
Scrape cytology result of Patient 2 Scrape cytology revealed atypical cells with nuclear atypia and hyperchromatic findings (Papanicolaou staining, original magnification ×40).

The lesion exhibited an induration in consistency accompanied by rough-surfaced mucosa without tenderness on palpation. There was no evident salivary flow from the sublingual caruncle. He did not have difficulty moving his tongue. The panoramic X-ray did not show any bone loss around the lesion (Figure 6B). Scrape cytology unveiled a small proportion of atypical cells with nuclear atypia and hyperchromatic findings (Figure 7).

**Figure 8:**
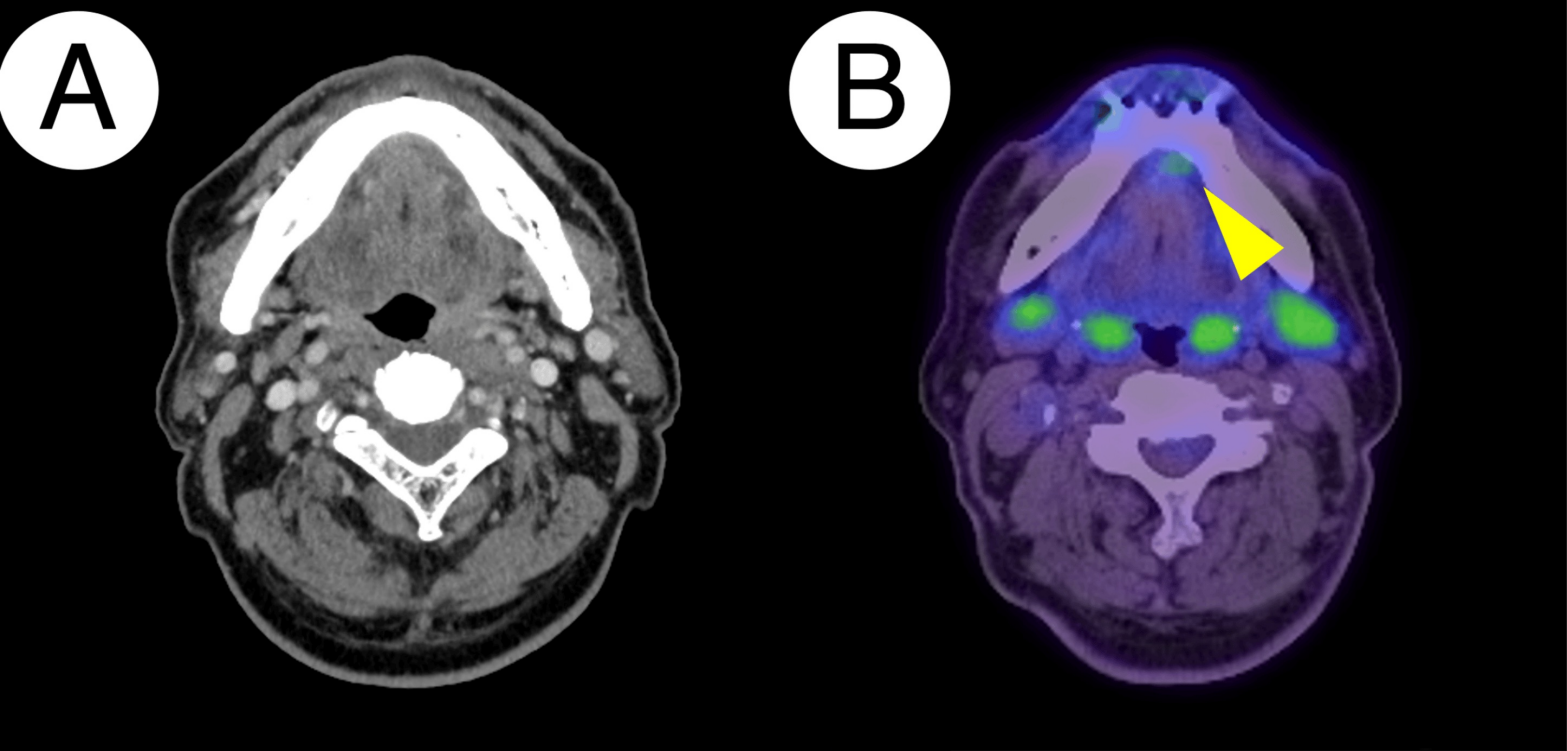
CT and PET images of Patient 2 Contrast-enhanced CT showed no apparent enhancing mass lesion around the sublingual caruncle nor apparent lymph node enlargement (A). PET depicted a hypermetabolic lesion (arrowhead) with a standard uptake value maximum of 3.217 (B). CT: computed tomography, PET: positron emission tomography

Contrast-enhanced CT depicted a mass on the ventral side of the lingual frenulum without apparent infiltration into the floor of the mouth or into the alveolar bone, along with no significant lymph node enlargement on each side of the submandibular region (Figure 8A). Ultrasonography revealed enlarged lymph nodes with well-defined and smooth margins: 8.9 × 7.3 × 5.6 mm (right level ⅠB), 13.5 × 9.6 × 3.9 mm (right level ⅡA), 7.8 × 6.7 × 3.5 mm (left level ⅠB), and 14.5 × 4.4 × 3.4 mm (left level ⅡA), respectively. Color Doppler imaging demonstrated blood flow signals corresponding to the lymphatic hilum, revealing that there were no supportive findings of lymph node metastasis. PET/CT presented increased FDG uptake around the lesion with an SUVmax of 3.217 (Figure 8B).

**Figure 9:**
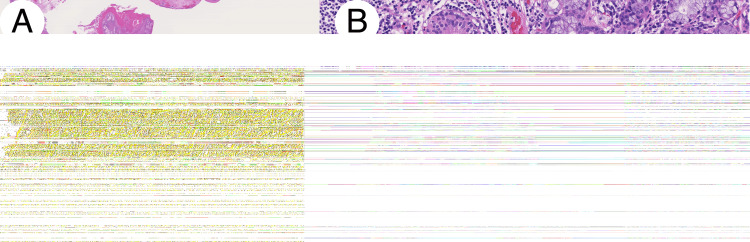
Pathological findings of Patient 2 Pathological examination revealed an increased mucous acinus and ductal proliferation accompanied by ductal dilatation and extensive infiltration of inflammatory cells. Loupe image of the lesion (A). High magnification of the lesion (original magnification ×200).

The initial clinical diagnosis was squamous cell carcinoma in the sublingual caruncle. Based on the examinations above and the mild increase in its size during the examinations, the decision was made to perform surgery under general anesthesia. One month after his first visit to our department, he underwent surgical resection with a 10 mm safety margin from the area of induration. The extent of resection included a part of the genioglossus muscle and the area above the sublingual gland, extending subperiosteally to the lingual side of the alveolar bone. Pathological examination revealed an increased number of mucous acini with standard ductal structure, along with squamous metaplasia of the ductal epithelium and bacterial aggregations in some ducts, accompanied by abscess formation and inflammatory cell infiltration surrounding the ducts, resulting in a diagnosis of AH with sialadenitis (Figure 9).

Postoperatively, he did not experience any complications or pain. Three months after the surgery, he is now being monitored regularly every six months without any signs of recurrence.

## Discussion

This case series highlighted AH of a minor salivary gland developing in a unique site, the buccal mucosa and sublingual caruncle, accompanied by inflammatory changes. AH generally arises from the oral mucosa, where minor salivary glands are densely distributed, such as the palate, buccal mucosa, and upper and lower lingual mucosa [1]. AH tends to develop in males predominantly between the 40s and 50s [8,9]. Its etiology remains unclear; however, chronic local trauma, especially an ill-fitting denture, is reported to contribute to the onset of the lesion [2]. Additionally, a previous report has demonstrated that a genetic factor, specifically a translocation at the t(2;14)(q21;q22) breakpoints, is also a potential factor in the development of AH [10]. Nonetheless, since there are some non-traumatic AH patients, the exact risk factors are still undefined. In our case series, Patient 1 experienced discomfort around the buccal lesion while gargling during her regular monitoring. Although she did not have any opportunistic factors such as denture or oral appliance wearing, her poor oral hygiene might have contributed to triggering inflammation of the lesion, resulting in her diagnosis of sialadenitis.

Diagnosis of a tumorous lesion in the oral cavity is generally challenging. Especially those lesions arising from the floor of the mouth and buccal mucosa are highly required to be distinguished from malignant tumors. Regarding tumors in the floor of the mouth, a previous paper reported that benign lesions account for 65% (2,613 cases) and malignant tumors for 35% (1,403 cases) in the Latin American population [11]. In addition, the paper also demonstrated that age over 60 was statistically associated with lesions arising from the floor of the mouth, diagnosed as oral leukoplakia and oral squamous cell carcinoma, respectively (p<0.0001 in the Chi-square test) [11]. Regarding tumors in the buccal mucosa, a previous study reported that approximately 10% (21 cases) of the lesions were benign tumors.

In comparison, 0.5% (1 case) of the 212 cases with oral cavity lesions were malignant tumors [12]. Oral squamous cell carcinoma (SCC) generally presents with an invasive growth pattern; however, malignant salivary gland tumors usually exhibit an indolent pattern. Hence, pathological examination using tumor-specific biomarkers, such as NR4A3, is crucial for diagnosing lesions with a latent phenotype [13]​​​​. Regarding AH, although there is no specific marker for AH, previous papers have performed some histochemical staining, including mucicarmine, periodic acid-Schiff (PAS), Ag nucleolar organizer regions (NOR) staining, a staining with silver that binds to NORs in a cellular nucleolus, and immunohistochemical (IHC) staining with Ki-67 for those patients. Generally, mucicarmine and PAS staining are performed to evaluate the distribution of glycogen and mucopolysaccharides, whose findings help distinguish hyperplasia from malignancies [14]​​​​​​.

Additionally, the mean Ag NOR counts also provide a clue to distinguish between normal salivary tissue, benign salivary gland tumors, chronic sialadenitis, and malignant salivary gland tumors [8]​​​​​​​​​​​​​​​​​. Moreover, the Ki-67 index, a marker for cellular proliferation activity, tends to show higher levels in malignant salivary gland tumors, including AdCC, than in normal salivary glands and AH of the salivary gland [8]. Although these histochemical and IHC analyses might not provide a specific pattern of AH itself, pathological analysis should play a significant role in distinguishing between specific types of malignant salivary gland tumors and others. Notably, these markers are not always reliable in distinguishing low-grade malignancies, which typically exhibit distinct staining profiles. In addition, AH sometimes presents with similar pathological findings to those of microepidermoid carcinoma, the most common malignant salivary gland tumor [15]. Generally, mucoepidermoid carcinoma consists of three cell types, including mucous cells, epidermoid cells, and intermediate cells, and its subtype that displays pathological findings with prominent mucous cells requires differential diagnosis with AH. In our patients, both cases exhibited normal glandular architecture without cell atypia in the resected specimens, which enabled us to distinguish them from malignant tumors. Accordingly, pathological examination in the early period should be considered. Moreover, our patients did not display robust growth, and the lesions were relatively small; hence, they underwent excisional biopsies as complete resections.

Regarding our pathological examinations, scrape cytology revealed atypical cells in both patients; however, specimens obtained by surgical resection did not display similar findings. This discrepancy could be explained by the sample limitation that scrape cytology captured the superficial stratified epithelium and did not include the lesion itself. For the cytology examinations, our patients were determined to undergo scrape cytology rather than fine-needle aspiration cytology because both lesions developed from a mucosal epithelial origin, not from a submucosal origin. Eventually, as described above, given that both specimens showed normal glandular structure without cytologic atypia accompanied by infiltration of inflammatory cells, further histochemical and IHC staining was not performed this time.

Definitive treatment for patients with AH is surgical resection. AH sometimes presents with similar manifestations of a malignant tumor. Hence, surgical resection with an adequate safety margin is warranted. Previously, the National Comprehensive Cancer Network guidelines recommended a surgical safety margin of at least 5 mm in oral SCCs [16]. On the other hand, there is still no consensus on determining the recommended safety margin for malignant salivary gland tumors due to their rarity [17]​​​​​​​​​​​​. A recent paper proposed that the safety margin of <5 mm for malignant salivary gland tumors would be sufficient since malignant salivary gland tumors with low-grade generally tend to show low infiltrative properties compared to those of oral SCC [17]. Notably, another paper demonstrated that low- to intermediate-grade malignant salivary gland tumors with a safety margin of less than 1 mm have similar local recurrence rates compared to those with safety margins larger than 1 mm [18]. In our case, Patients 1 and 2 underwent surgical resection with 5 mm and 10 mm, respectively, due to suspicion of malignancies. During follow-up, Patient 1 presented with mild trismus, whereas Patient 2 experienced no apparent postoperative complications. These findings support the safety and effectiveness of the surgical resection with appropriate safety margins in AH cases.

Our patients were eventually managed as having AH with inflammation; however, several limitations need to be addressed. Firstly, artifacts caused by dental metal hampered the accurate evaluation of the lesion by imaging modalities. In our report, each patient underwent examination of their lesions using several imaging modalities, including CT, MRI, and PET. These imaging modalities ruled out the potential for lymph node metastasis in their head and neck region; however, the small lesion size, less than 10 mm, impeded an accurate evaluation of its nature. Thus far, previous papers have demonstrated the availability of PET/CT in the initial diagnosis of malignant salivary gland tumors. Especially volumetric parameters, such as median metabolic tumor volume and total lesion glycolysis, offer clues for distinguishing between benign and malignant salivary gland tumors [19]​​​​​​​.

Furthermore, a recent paper proposed that MRI offers various advantages over ultrasound, including excellent contrast resolution between normal tissues and lesions, as well as accessibility to deep tissue structures [20]. However, it is noteworthy that lesions <10 mm in size would be difficult to evaluate by MRI accurately due to limited spatial resolution and decreased signal-to-noise ratio [20]. Secondly, histochemical and IHC analyses were not performed on our patient during their management because both specimens showed typical histological findings of AH. However, these staining methods might have provided additional information by identifying features specific to AH, helping distinguish it from malignant salivary gland tumors and potentially guiding the follow-up period. Therefore, addressing these limitations can lead to a better understanding of its characteristics and diagnosis.

## Conclusions

This case series demonstrates that AH can develop in unusual sites, emphasizing the importance of differential diagnosis, particularly in distinguishing AH from malignant tumors. Imaging modalities have some limitations in detecting small lesions, thereby enhancing the value of histopathological examination. As demonstrated in this case series, complete surgical excision is an effective treatment for AH.

## References

[1] Raju PR, Manyam R, Ahalya P (2023). Adenomatoid hyperplasia of minor salivary glands: a case report in an unusual site. Int J Surg Case Rep.

[2] Barrett A, Speight P (1995). Adenomatoid hyperplasia of oral minor salivary glands. Oral Surg Oral Med Oral Pathol Oral Radiol.

[3] Bryant C, Manisali M, Barrett AW (1996). Adenomatoid hyperplasia of palatal minor salivary glands. J Laryngol Otol.

[4] Scully C, Eveson JW, Richards A (1992). Adenomatoid hyperplasia in the palate: another sheep in wolf's clothing. Br Dent J.

[5] Alsanie I, Rajab S, Cottom H (2022). Distribution and frequency of salivary gland tumours: an international multicenter study. Head Neck Pathol.

[6] Sardar MA, Ganvir S, Hazarey V (2018). A demographic study of salivary gland tumors. SRM J Res Dent Sci.

[7] Brannon RB, Houston GD, Meader CL (1985). Adenomatoid hyperplasia of mucous salivary glands: a case involving the retromolar area. Oral Surg Oral Med Oral Pathol.

[8] Braz GL, Vasconcelos AC, Gomes AP, Calderipe CB, Soares AC (2025). Adenomatoid hyperplasia of minor salivary glands: a systematic review. Oral Surg Oral Med Oral Pathol Oral Radiol.

[9] Altındağ A, Bozkurt P, Bilecenoğlu B, Orhan K (2021). Adenomatoid hyperplasia of the oral cavity: a diagnostic dilemma. Eur Ann Dent Sci.

[10] Manor E, Sinelnikov I, Brennan PA, Bodner L (2013). Chromosomal aberrations in adenomatoid hyperplasia of palatal minor salivary gland. Br J Oral Maxillofac Surg.

[11] Costa AM, Pontes FS, Souza LL (2021). What is the frequency of floor of the mouth lesions? A descritive study of 4,016 cases. Med Oral Patol Oral Cir Bucal.

[12] Błochowiak K, Farynowska J, Sokalski J, Wyganowska-Świątkowska M, Witmanowski H (2019). Benign tumours and tumour-like lesions in the oral cavity: a retrospective analysis. Postepy Dermatol Alergol.

[13] Haller F, Skálová A, Ihrler S (2019). Nuclear NR4A3 immunostaining is a specific and sensitive novel marker for acinic cell carcinoma of the salivary glands. Am J Surg Pathol.

[14] Mahamad Apandi NI, Chan SW, Toh YF (2024). Differential expression of mucin in salivary gland tumours. Medicina (Kaunas).

[15] Peraza A, Gómez R, Beltran J, Amarista FJ (2020). Mucoepidermoid carcinoma. An update and review of the literature. J Stomatol Oral Maxillofac Surg.

[16] Gill A, Vasan N, Givi B, Joshi A (2018). Ahns series: do you know your guidelines? Evidence-based management of oral cavity cancers. Head Neck.

[17] Geiger JL, Ismaila N, Beadle B (2021). Management of salivary gland malignancy: ASCO guideline. J Clin Oncol.

[18] Sajisevi M, Nguyen K, Callas P (2024). Oncologic safety of close margins in patients with low- to intermediate-grade major salivary gland carcinoma. JAMA Otolaryngol Head Neck Surg.

[19] Gencturk M, Ozturk K, Koksel Y, Li F, Cayci Z (2019). Pretreatment quantitative (18)F-FDG PET/CT parameters as a predictor of survival in adenoid cystic carcinoma of the salivary glands. Clin Imaging.

[20] Varoquaux A, Fakhry N, Baujat B (2024). Diagnostic imaging of salivary gland cancers: REFCOR recommendations by the formal consensus method. Eur Ann Otorhinolaryngol Head Neck Dis.

